# Supplementary material for: Light-insensitive organic solar-powered amplifiers
Source: Nat Commun. 2025 Nov 27;16:10640. doi: 10.1038/s41467-025-65640-z (PMC12660383; doi:10.1038/s41467-025-65640-z)
Supplement: Supplementary file 1 — Supplementary Information [file 41467_2025_65640_MOESM1_ESM.pdf]

Supplementary Information

Light-Insensitive Organic Solar-Powered Amplifiers

Qiang Wu[1#], Shijie Wang[1#], Wei Gao[2#], Zeng Chen[3], Jun Tao[4], Xinyue Song[5], Sen Yan[5], Haiming Zhu[3], Ke Zhou[1], Long Jiang[6*], Xiaomin Xu[4*], Alex K.-Y. Jen[2*], Wei Ma[1*]

[1]State Key Laboratory for Mechanical Behavior of Materials, Xi'an Jiaotong University, Xi'an, 710049, China
[2]Department of Materials Science and Engineering, City University of Hong Kong, Kowloon, 999077, Hong Kong
[3]State Key Laboratory of Modern Optical Instrumentation, Key Laboratory of Excited State Materials of Zhejiang Province, Department of Chemistry, Zhejiang University, Hangzhou, 310027, China
[4]Institute of Materials Research, Shenzhen International Graduate School, Tsinghua University, Shenzhen, 518055, China
[5]School of Information and Communications Engineering, Xi'an Jiaotong University, Xi'an, 710049, China
[6]State Key Laboratory of Oil and Gas Equipment CNPC Tubular Goods Research Institute, Xi'an,710077, China

[#]These authors contributed equally.
E-mail: jianglong003@cnpc.com.cn; xu.xiaomin@sz.tsinghua.edu.cn; alexjen@cityu.edu.hk; msewma@xjtu.edu.cn

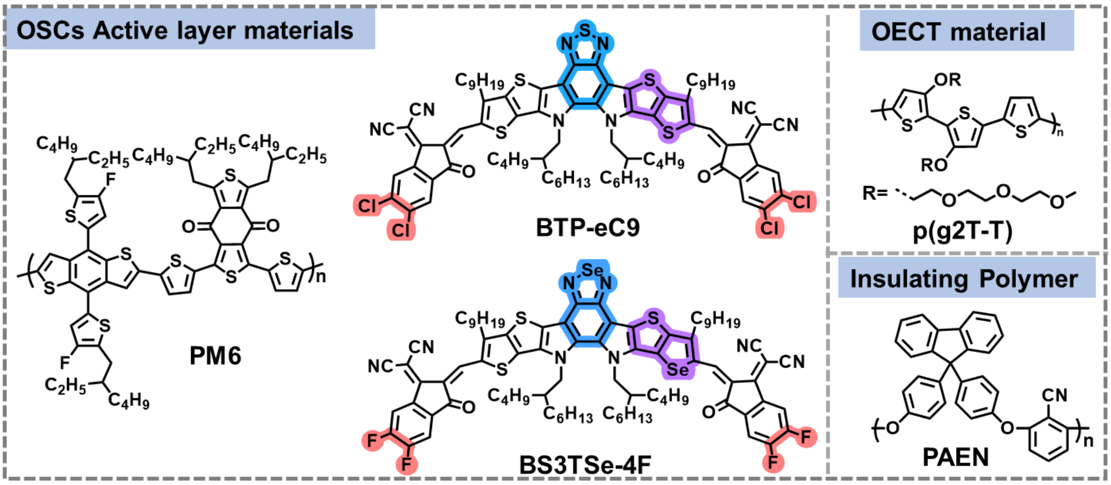

Supplementary Fig. 1 | Molecular structures. Chemical structures of the active materials of OSCs and OECT material as well as insulation polymer material investigated in this work.

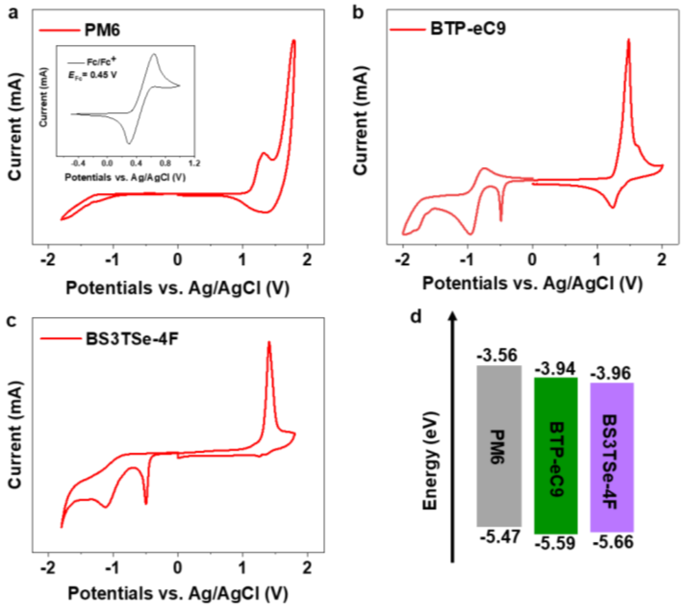

Supplementary Fig. 2 | Energy level alignments of different materials. Cyclic voltammogram (CV) curves of **a** PM6, **b** BTP-eC9 and **c** BS3TSe-4F films on glassy carbon electrode in 0.1 mol L^{-1} Bu_4NPF_6 acetonitrile solution. **d** Energy level alignments of relevant components of OSCs devices.

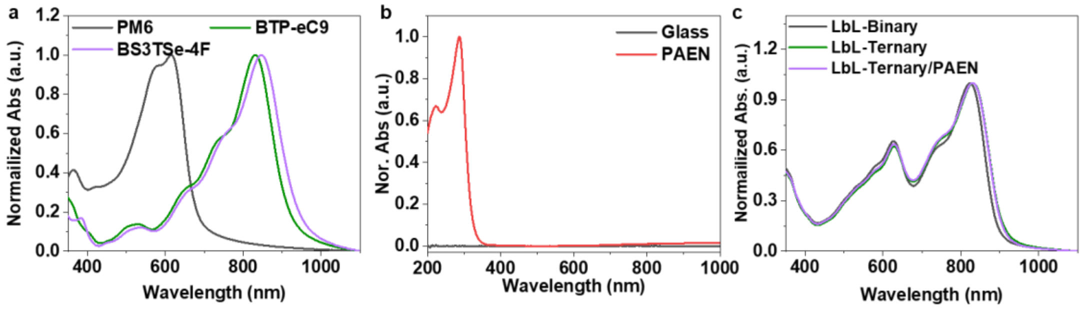

Supplementary Fig. 3 | Normalized absorption spectra of different materials. a Normalized absorption spectra of the pristine films of active layer materials. **b** Normalized absorption spectra of the PAEN and glass substrate. **c** Normalized absorption spectra of the different film devices based on sequential layer-by-layer processing technology.

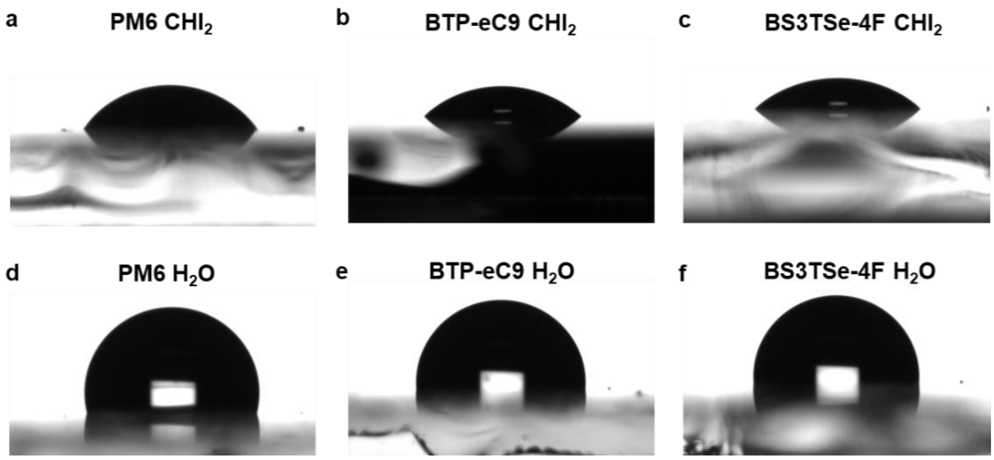

Supplementary Fig. 4 | Contact angel of the different films. Photographs of CHI_2 **a-c** and H_2O **d-f** droplets on the top surfaces of PM6, BTP-eC9 and BS3TSe-4F film.

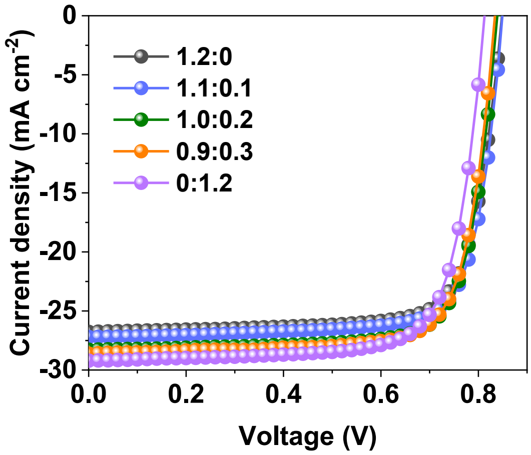

Supplementary Fig. 5 | *J-V* curves of the binary and the optimized ternary OPV cells under AM 1.5G, 100 mA cm^{-2}.

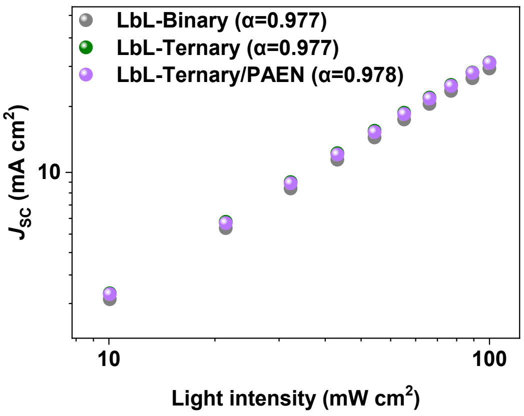

Supplementary Fig. 6 | Measurement of J_{SC} versus light intensity for the devices.

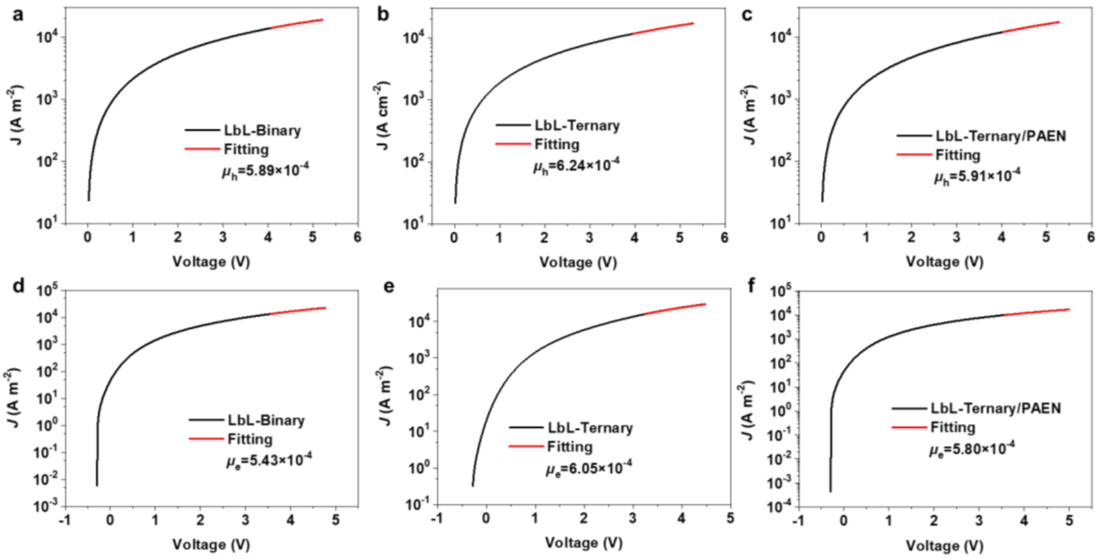

Supplementary Fig. 7 | Electron- or hole-only mobility of different films. The dark J-V characteristics of electron-only mobility of **a** the LbL-Binary device, **b** the LbL-Ternary device and **c** LbL-Ternary/PAEN device. The dark J-V characteristics of hole-only mobility of **d** the LbL-Binary device, **e** the LbL-Ternary device and **f** LbL-Ternary/PAEN device. The red lines represent the best fitting using the SCLC model.

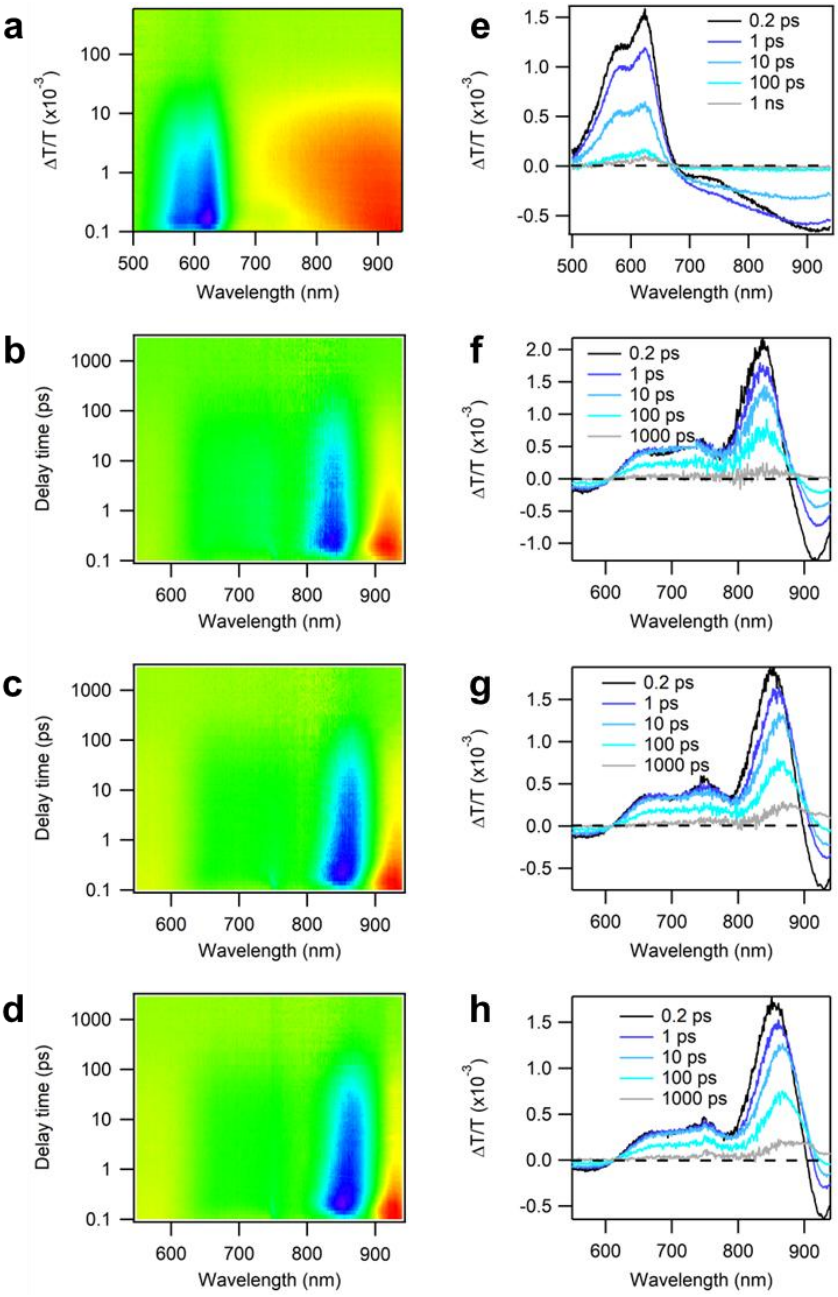

Supplementary Fig. 8 | TA measurement. 2D color plots of TA spectra of **a** the pure PM6 film, **b** the pure BTP-eC9 film, **c** the pure BS3TSe-4F and **d** the blend BTP-eC9:BS3TSe-4F. TA spectra of **e** the pure PM6 film, **f** the pure BTP-eC9 film, **g** the pure BS3TSe-4F and **h** the blend BTP-eC9:BS3TSe-4F at indicated delay times.

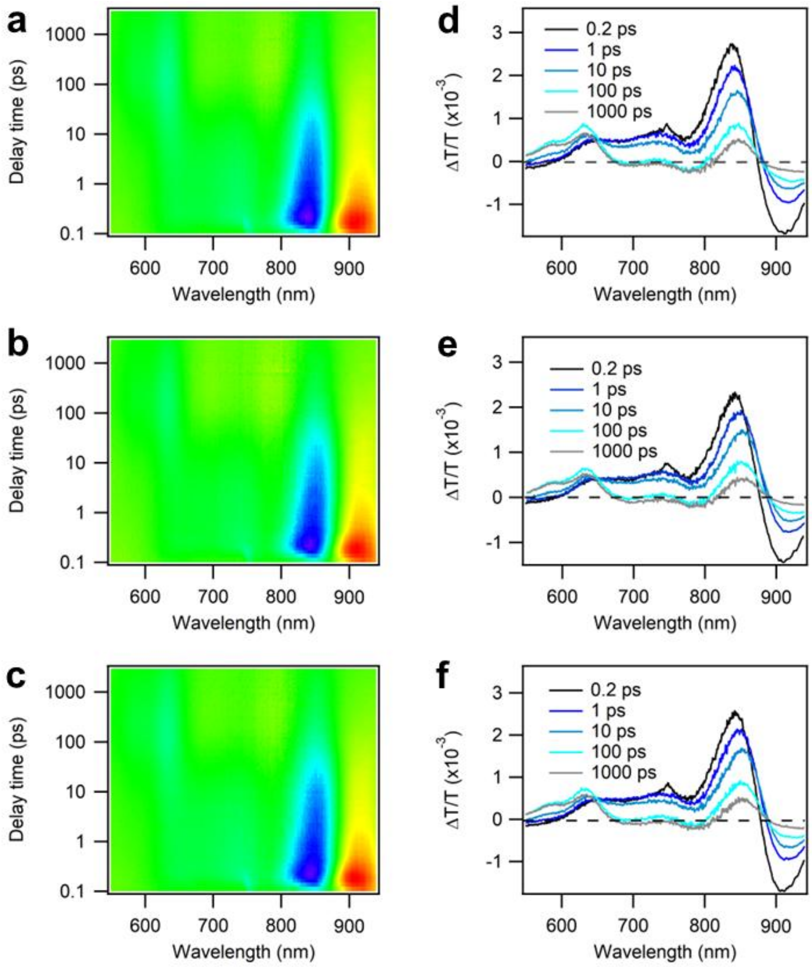

Supplementary Fig. 9 | TA measurement. 2D color plots of TA spectra of **a** the LbL-Binary film, **b** the LbL-Ternary film and **c** LbL-Ternary/PAEN film. TA spectra of **d** the LbL-Binary film, **e** LbL-Ternary film and **f** LbL-Ternary/PAEN film at indicated delay times.

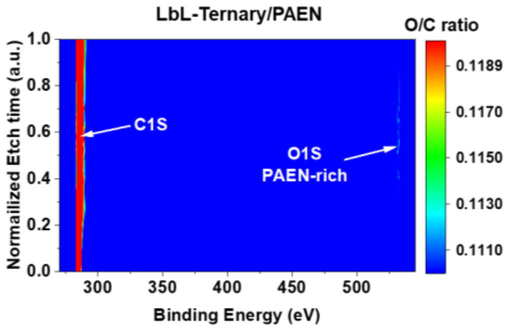

Supplementary Fig. 10 | Depth analysis XPS profiles for the LbL-Ternary-PAEN film (normalized by the intensity of C atom).

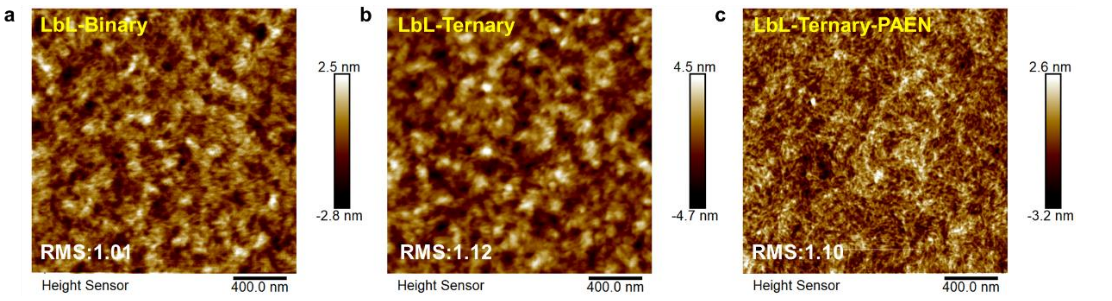

Supplementary Fig. 11 | AFM measurement. AFM phase images of **a** LbL-Binary **b** LbL-Ternary and **c** LbL-Ternary/PAEN films.

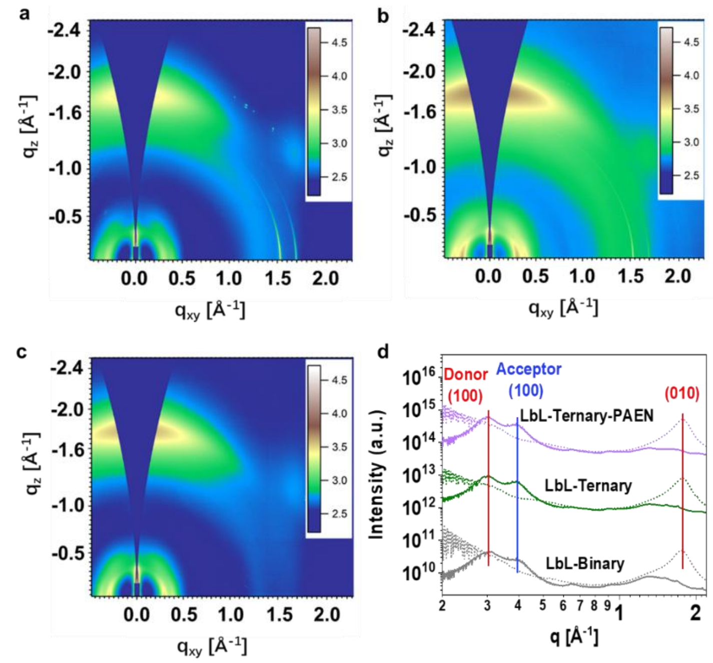

Supplementary Fig. 12 | GIWAXS measurements. 2D GIWAXS profiles of (a) LbL-Binary (b) LbL-Ternary and (c) LbL-Ternary/PAEN films. (d) Scattering profiles of IP and OOP for PM6 and PM6:PYT blends. (The Solid lines are in-plane (IP) orientations; The dotted line is the out-of-plane (OOP) orientation.)

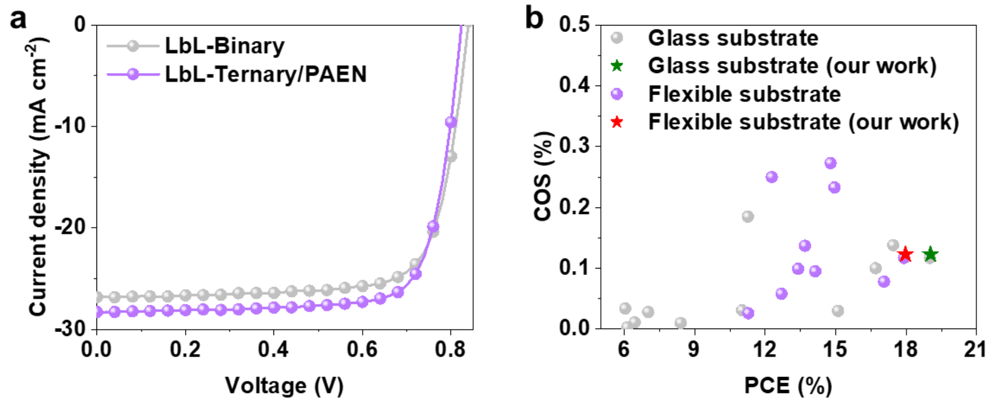

Supplementary Fig. 13 | Performance of flexible OSCs. a The *J*-*V* curves of the LbL-Binary and LbL-Ternary-PAEN devices based on PET/ITO substrate measured under 1

sun illumination. **b** The plots of the PCE versus COS for the efficient OSCs reported in the literature.

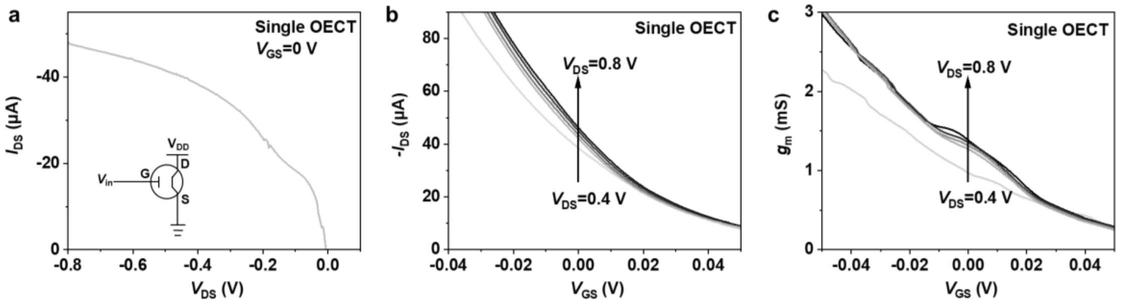

Supplementary Fig. 14 | Output curve and Transfer curves of single OECT. Output curve **a**, Transfer curves **b** and associated transconductance **c** of the Source Meter Unit (SMU) powered amplifier at different bias voltage.

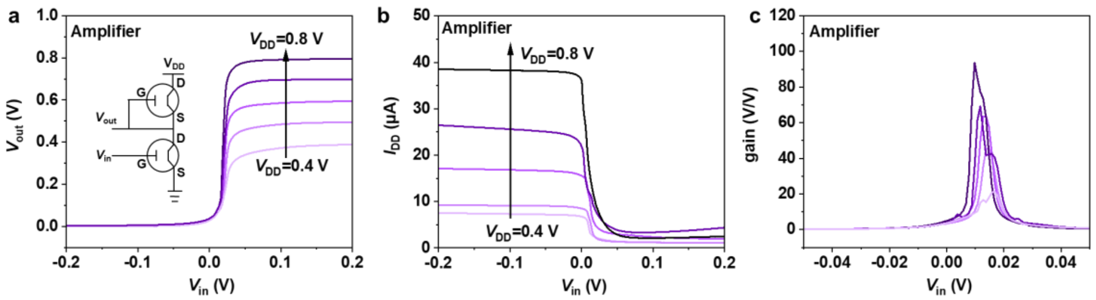

Supplementary Fig. 15 | Output curve and Transfer curves of amplifier. Output curve (**a**) and Transfer curves (**b**) of the Source Meter Unit (SMU) powered amplifier at different bias voltage. **c** Related Gain of amplifier at different bias voltage.

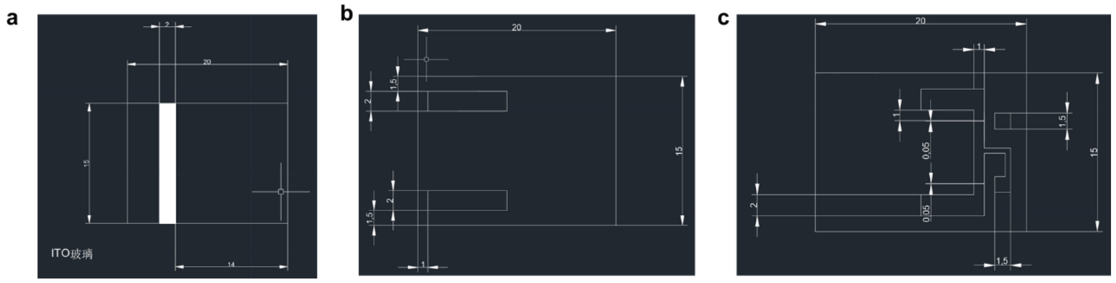

Supplementary Fig. 16 | Design drawings of integrated devices. a The figure of the pre-patterned ITO coated glass substrates (The white area is the ITO area). **b** The designed figures of the mask to evaporate the electrode based on the OSCs. **c** The designed figures of the mask to evaporate the electrode based on the amplifier (mm).

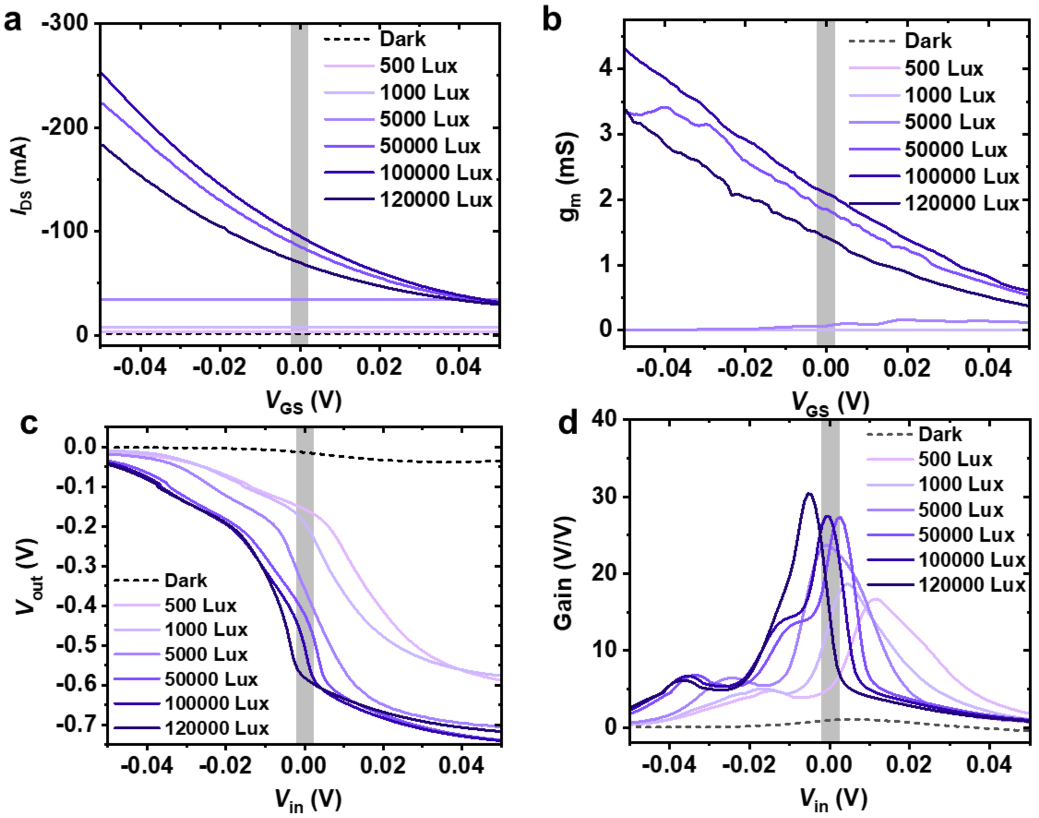

Supplementary Fig. 17 | Transfer curves of the integrated device at different light intensity. Transfer curves **a** and related transconductance **b** of the OSCs powered OECT at different light intensity. Transfer curves **c** and related gain (**d**) of the OSCs powered amplifier at different light intensity.

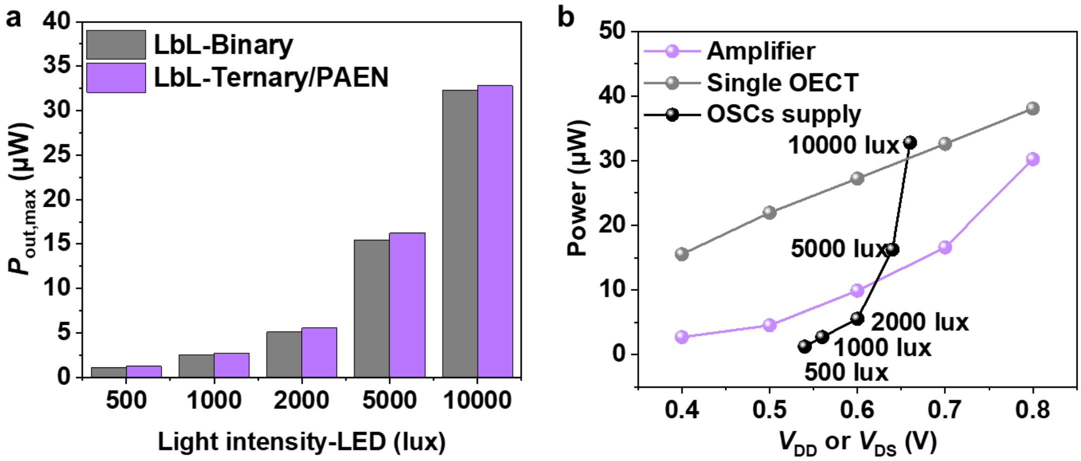

Supplementary Fig. 18 | Performance of different devices. a The $P_{out,max}$ of LbL-Binary and LbL-Ternary/PAEN devices. **b** Minimum power of single OECT and amplifier at different bias pressures versus maximum output power of OSCs under different light intensities.

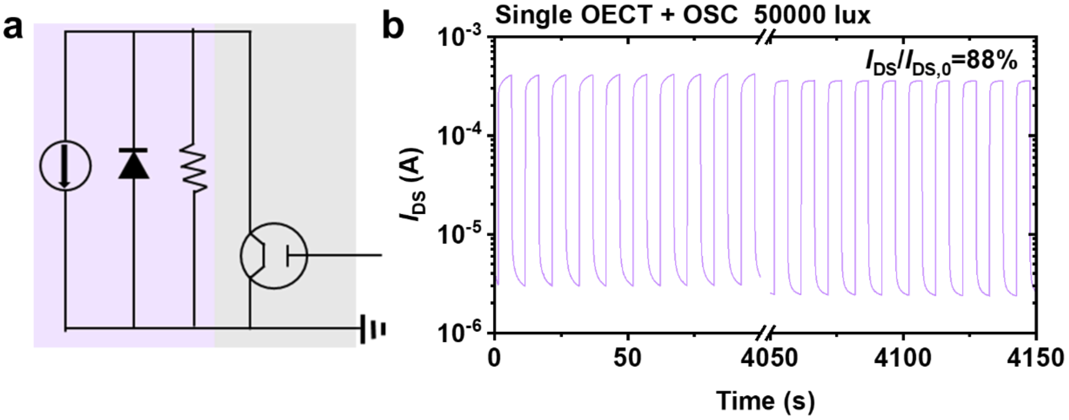

Supplementary Fig. 19 | Operation stability of an OSC powered single OECT. a Circuit diagram used for the test. **b** Operation stability of the OSCs powered single OECT with an input frequency of 1 Hz. The light intensity used here is 50,000 lux.

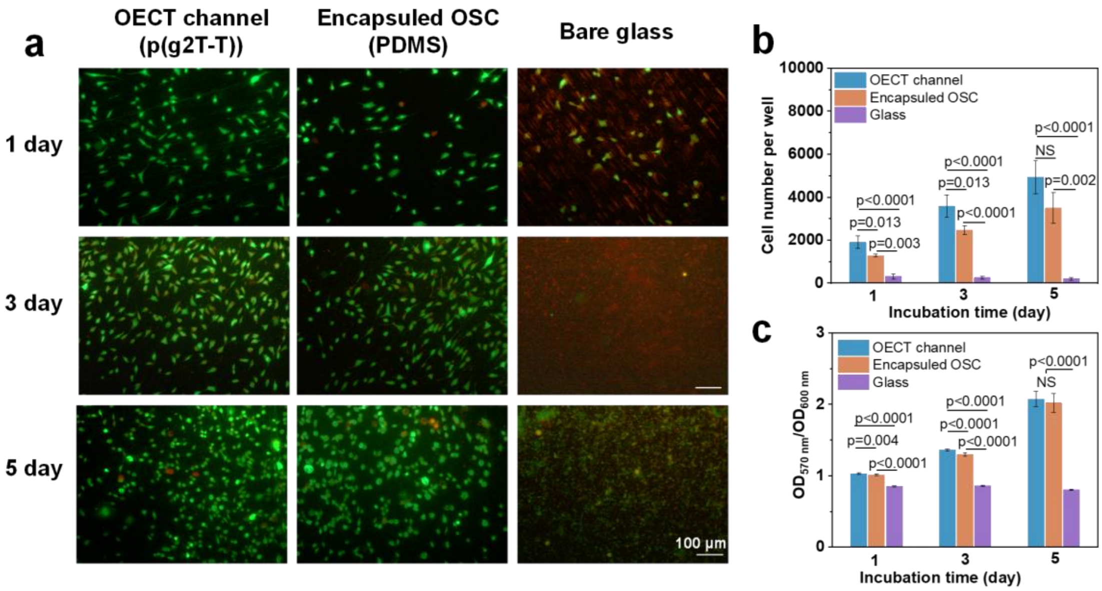

Supplementary Fig. 20 | Biocompatibility test of different parts in the integrated device. a Fluorescence microscope of the PC-12 cells cultured on different device-cell interfaces. Cell density **c** and 3-(4,5-Dimethylthiazol-2-yl)-2,5-diphenyltetrazolium bromide (MTT) tested mitochondrial activities **d** on different device-cell interfaces.

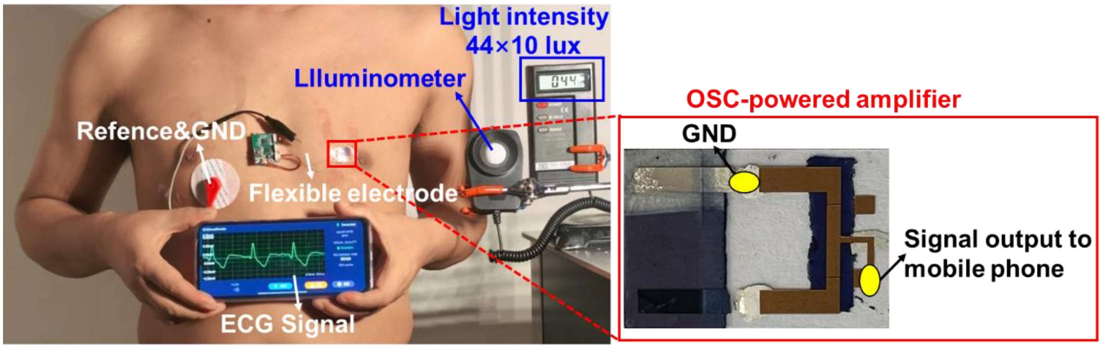

Supplementary Fig. 21 | The capture of Video S1 that shows the ECG monitoring at500 lux right panel shows the different components of integrated devices.

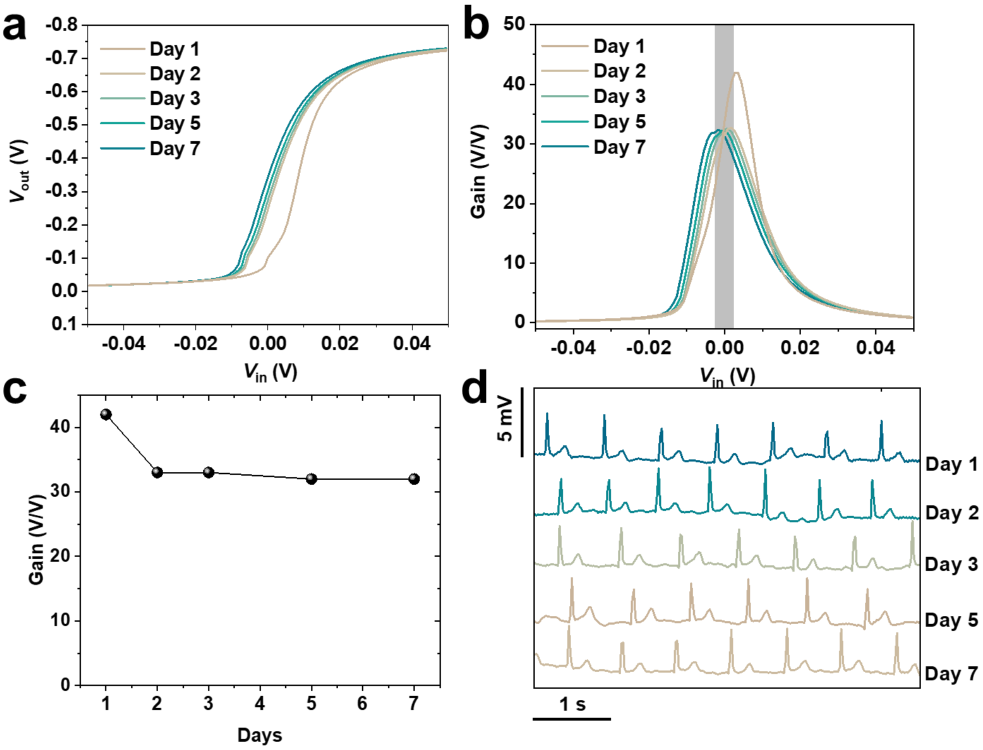

Supplementary Fig. 22 | Long-term stability of an on-body champion OSC powered amplifier in air atmosphere (light intensity: 5,000 lux). Output curve **a** and gain curve **b** of the OSC powered amplifier after different storage time. Inset shows the on-body OSC powered amplifier used for test. **c** Plot of gain values versus storage time. **d** ECG signals recorded by OSC powered amplifier on different days.

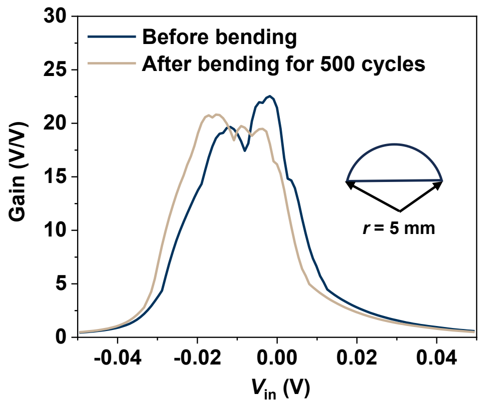

Supplementary Fig. 23 | Mechanical stability of the OSC powered amplifier.

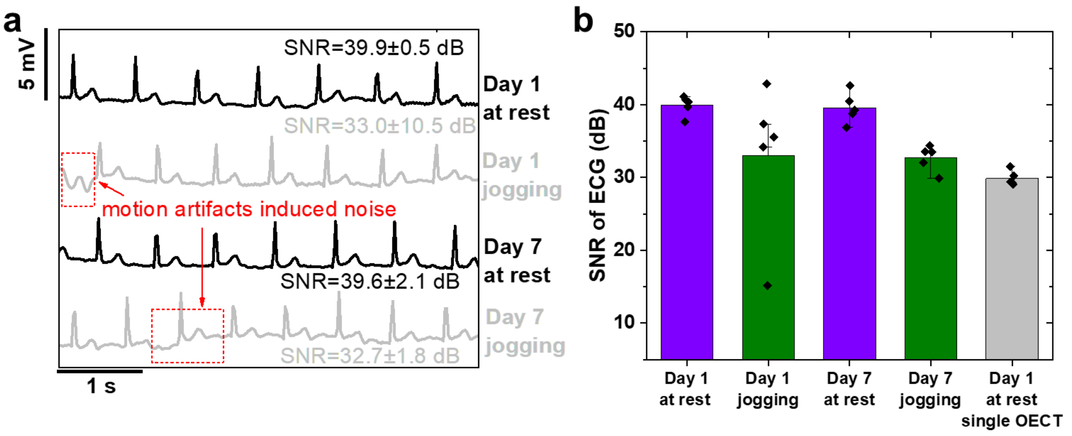

Supplementary Fig. 24 | Noise analysis of signals recorded by integrated devices. **a** ECG signals recorded in rest or jogging state before and after one week of wearing. **b** Signal-to-noise ratio (SNR) of ECG signals recorded at different state before and after one week of wearing.

Supplementary Table 1 | Contact angles of H_2O and CHI_2 and their parameters for active layer materials

Materials	Contact angle (°, CH_2I_2)	Contact angle (°, H_2O)	Surface Energy (mN m^{-1})	$\chi_{(D,A)}$	$\chi_{(A,A)}$
PM6	56.2	104.1	30.82		
BTP-eC9	43.1	99.1	38.09	0.385	0.0002
BS3TSe-4F	43.4	100.9	37.9	0.366	

Supplementary Table 2 | Photovoltaic parameters of the binary and the optimized ternary OPV cells.

BTP-eC9:BS3TSe-4F	V_{OC} (V)	J_{SC} (mA cm-2)	FF (%)	PCE (%)
1.2:0	0.849	26.73	76.99	17.47 (17.34±0.16)
1.1:0.1	0.849	27.21	77.45	17.90 (17.58±0.32)
1.0:0.2	0.843	28.27	77.15	18.38 (18.11±0.25)
0.9:0.3	0.834	28.62	76.68	18.30 (18.22±0.11)
0:1.2	0.812	29.20	75.34	17.88 (17.55±0.31)

Supplementary Table 3 | Photovoltaic parameters of the binary and the optimized ternary OPV cells.

System	Light source (LED)	P_{in} (mW cm^{-2})	V_{OC} (V)	J_{SC} (μA cm^{-2})	FF (%)	P_{out} (mWcm^{-2})
LbL-Binary	500 lux	210.95	0.660	73.94	54.10	26.40 (25.65±0.67)
	1000 lux	428.43	0.689	145.43	62.88	63.01(63.68±1.05)
	2000 lux	813.36	0.706	274.36	65.70	127.26 (123.76±7.19)
LbL-Ternary/PAEN	500 lux	210.95	0.658	78.97	59.35	30.84 (30.87±1.33)
	1000 lux	428.43	0.682	154.00	64.61	67.86 (69.23±1.60)
	2000 lux	813.36	0.705	279.48	70.12	138.16 (138.29±0.15)

Supplementary Table 4 | The physic properties of the different devices.

Sample	Quenching efficiency (PL)	Charge dissociation probability	μ_h (cm^2 V^{-1} s^{-1})	μ_e (cm^2 V^{-1} s^{-1})	μ_h/μ_e
LbL-Binary	81.39%	98.17%	5.89×10^{-4}	5.43×10^{-4}	1.08
LbL-Ternary	86.89%	98.21%	6.24×10^{-4}	6.05×10^{-4}	1.03
LbL-Ternary/PAEN	86.44%	98.37%	5.91×10^{-4}	5.80×10^{-4}	1.02

Supplementary Table 5 | The kinetics parameters of the HT processes of the three films, calculated from femtosecond transient absorption spectroscopy.

Sample	A_1	τ_1 (ps)	A_2	τ_2 (ps)
LbL-Binary	0.33	0.21	0.67	27.8
LbL-Ternary	0.34	0.18	0.66	25.8
LbL-Ternary/PAEN	0.40	0.15	0.60	27.2

Supplementary Table 6| A summary of fitted crystal parameters of the corresponding devices.

Sample	IP_{Donor} (100)		$IP_{Acceptor}$ (100)		OOP (010)			
	Location ($Å^{-1}$)	d-spacing (Å)	Location ($Å^{-1}$)	d-spacing (Å)	Location ($Å^{-1}$)	d-spacing (Å)	FWHM	CCL (Å)
LbL-Binary	0.303	20.74	0.391	16.07	1.761	3.57	0.199	31.57
LbL-Ternary	0.303	20.74	0.391	16.07	1.775	3.54	0.187	33.60
LbL-Ternary-PAEN	0.303	20.74	0.391	16.07	1.778	3.53	0.187	33.60

Supplementary Table 7 | Mechanical properties of the LbL-Binary and LbL-Ternary-PAEN films from strain-stress curves.

Organic layer	COS	Toughness (KJ m^{-3})
LbL-Binary	0.070	1.78
LbL-Ternary/PAEN	0.123	3.34

Supplementary Table 8 | Photovoltaic parameters of the LbL-Binary and the LbL-Ternary/PAEN devices based on PET/ITO subitrate.

**Sample**	V_{OC} **(V)**	J_{SC} **(mA cm^{-2})**	**FF (%)**	**PCE[a] (%)**
LbL-Binary	0.839	26.82	75.70	17.03 (16.94±0.13)
LbL-Ternary/PAEN	0.823	28.31	77.11	17.97 (17.61±0.31)

a) The average PCE values with standard deviations were obtained from 6 individual devices.

Supplementary Table 9 | The corresponding PCE and COS of polymer:small micromolecule acceptors based on glass or flexible substrate reported in literature.

Substrate	Active layer	PCE(%)	COS	Refence
Glass	PTB7-Th:PC71BM	8.40	0.01	1
Glass	PTB7-Th: PCBM	6.04	0.034	2
Glass	PTB7-Th: ITIC	6.44	0.011	2
Glass	PBDTTTPD:PCBM	6.12	0.003	3
Glass	PBDB-T: Y5-2BO	7.02	0.028	4
Glass	PM6:Y7	15.1	0.03	5
Glass	PM6-C5:Y7	16.7	0.1	5
Glass	PTB7-Th:FOIC	11.01	0.031	6
Glass	PTB7-Th/FOIC	11.26	0.185	6
Glass	PhAm5:Y7	17.45	0.138	7
Glass	D18:N3:DOY-C4	19.01	0.117	8
Glass	PM6/PAEN/BTP-eC9:BS3TSe-4F	19.04	0.123	Our work
PET	PM6:Y6:PAEF	12.28	0.25	9
PET	PM6:Y6	12.69	0.058	9
PET	PM6:Y6-BO-4Cl:PAE	13.40	0.099	10
PET	PM6:PBB1-F:Y6-BO-4Cl:PAE	14.78	0.273	10
PET	PM6:BTP-eC9:PAE	14.13	0.095	10
PET	PM6:PBB1-F:BTP-eC9:PAE	14.95	0.233	10
TPU	PM7:L8-BO	11.28	0.026	11
TPU	PM7-Thy10:L8-BO	13.69	0.137	11
PET	D18:N3	17.06	0.078	8
PET	D18:N3:DOY-C4	17.91	0.117	8
PET	PM6/BTP-eC9	17.03	0.070	Our work
PET	PM6/PAEN/BTP-eC9:BS3TSe-4F	17.97	0.123	Our work

Supplementary Table 10 | Signal-to-noise ratio (SNR) of the electrophysical signals recorded by OSC powered amplifier and single OECT.

Different electrophysical signals	Light intensity		
	500 lux	5,000 lux	50,000 lux
SNR of EMG (OSC powered single OECT)	21.1±4.3 dB	27.3±0.9 dB	35.0±2.8 dB
SNR of EMG (OSC powered amplifier)	41.7±1.6 dB	44.3±2.2 dB	44.5±2.9 dB
SNR of ECG (OSC powered single OECT)	29.6±2.4 dB	31.0±2.2 dB	34.1±4.9 dB
SNR of ECG (OSC powered amplifier)	43.0±4.0 dB	43.0±1.5 dB	43.9±3.7 dB
SNR of EOG (OSC powered single OECT)	20.9±4.5 dB	28.3±2.1 dB	42.7±1.9 dB
SNR of EOG (OSC powered amplifier)	50.0±0.7 dB	51.2±1.0 dB	54.0±4.7 dB

Refence:

1. Lee, W., Kim, J.-H., Kim, T., Kim, S., Lee, C., Kim, J.-S., Ahn, H., Kim, T.-S. & Kim, B. J. Mechanically robust and high-performance ternary solar cells combining the merits of all-polymer and fullerene blends. *J. Mater. Chem. A* **6**, 4494 (2018).
2. Choi, J., Kim, W., Kim, S., Kim, T.-S. & Kim, B. J. Influence of acceptor type and polymer molecular weight on the mechanical properties of polymer solar cells. *Chem. Mater.* **31**, 9057 (2019).
3. Kim, T., Kim, J. H., Kang, T. E., Lee, C., Kang, H., Shin, M., Wang, C., Ma, B., Jeong, U., Kim, T. S. & Kim, B. J. Flexible, highly efficient all-polymer solar cells. *Nat. Commun.* **6**, 8547 (2015).
4. Lee, J. W., Sun, C., Ma, B. S., Kim, H. J., Wang, C., Ryu, J. M., Lim, C., Kim, T. S., Kim, Y. H., Kwon, S. K. & Kim, B. J. Efficient, thermally stable, and mechanically robust all-polymer solar cells consisting of the same benzodithiophene unit-based polymer acceptor and donor with high molecular compatibility. *Adv. Energy Mater.* **11**, 2003367 (2020).
5. Lee, J. W., Jeong, D., Kim, D. J., Phan, T. N. L., Park, J. S., Kim, T. S. & Kim, B. J. Flexible-spacer incorporated polymer donors enable superior blend miscibility for high-performance and mechanically-robust polymer solar cells. *Energy Environ. Sci.* **14**, 4067-4076 (2021).
6. Wang, Y., Zhu, Q., Naveed, H. B., Zhao, H., Zhou, K. & Ma, W. Sequential blade-coated acceptor and donor enables simultaneous enhancement of efficiency, stability, and mechanical properties for organic solar cells. *Adv. Energy Mater.* **10**, 1903609 (2020).
7. Lee, J. W., Seo, S., Lee, S. W., Kim, G. U., Han, S., Phan, T. N. L., Lee, S., Li, S., Kim, T. S., Lee, J. Y. & Kim, B. J. Intrinsically stretchable, highly efficient organic solar cells enabled by polymer donors featuring hydrogen-bonding spacers. *Adv. Mater.* **34**, 2207544 (2022).
8. Ye, Q., Chen, Z., Yang, D., Song, W., Zhu, J., Yang, S., Ge, J., Chen, F. & Ge, Z. Ductile oligomeric acceptor-modified flexible organic solar cells show excellent mechanical robustness and near 18% efficiency. *Adv. Mater.* **35**, 2305562 (2023).
9. Han, J. H., Bao, F., Huang, D., Wang, X. C., Yang, C. M., Yang, R. Q., Jian, X. G., Wang, J. Y., Bao, X. C. & Chu, J. H. A universal method to enhance flexibility and stability of organic solar cells by constructing insulating matrices in active layers. *Adv. Funct. Mater.* **30**, 2003654 (2020).
10. Wang, J., Han, C., Han, J., Bi, F., Sun, X., Wen, S., Yang, C., Yang, C., Bao, X. & Chu, J. Synergetic strategy for highly efficient and super flexible thick-film organic solar cells. *Adv. Energy Mater.* **12**, 2201614 (2022).
11. Wan, Q., Seo, S., Lee, S. W., Lee, J., Jeon, H., Kim, T. S., Kim, B. J. & Thompson, B. C. High-performance intrinsically stretchable polymer solar cell with record efficiency and stretchability enabled by thymine-functionalized terpolymer. *J. Am. Chem. Soc.* **142**, 11914 (2023).
