# Supplementary material for: Light-insensitive organic solar-powered amplifiers
Source: Nat Commun. 2025 Nov 27;16:10640. doi: 10.1038/s41467-025-65640-z (PMC12660383; doi:10.1038/s41467-025-65640-z)
Supplement: Supplementary file 2 — Description of Additional Supplementary Information [file 41467_2025_65640_MOESM2_ESM.pdf]

**Description of Additional Supplementary Files**

File Name: Supplementary Movie 1
Description: Real-time ECG monitoring with the self-powered wearable biosensor under 500 lux illumination.

File Name: Supplementary Movie 2
Description: Real-time ECG monitoring with the self-powered wearable biosensor under 5000 lux illumination.
