# Supplementary material for: Light-insensitive organic solar-powered amplifiers
Source: Nat Commun. 2025 Nov 27;16:10640. doi: 10.1038/s41467-025-65640-z (PMC12660383; doi:10.1038/s41467-025-65640-z)
Supplement: Supplementary file 5 — Reporting Summary [file 41467_2025_65640_MOESM5_ESM.pdf]

nature portfolio

Corresponding author(s): Wei Ma

Last updated by author(s): 2025/10/14

Reporting Summary

Nature Portfolio wishes to improve the reproducibility of the work that we publish. This form provides structure for consistency and transparency in reporting. For further information on Nature Portfolio policies, see our Editorial Policies and the Editorial Policy Checklist.

Statistics

For all statistical analyses, confirm that the following items are present in the figure legend, table legend, main text, or Methods section.

n/a	Confirmed	
☐	☒	The exact sample size (*n*) for each experimental group/condition, given as a discrete number and unit of measurement
☐	☒	A statement on whether measurements were taken from distinct samples or whether the same sample was measured repeatedly
☒	☐	The statistical test(s) used AND whether they are one- or two-sided<br>*Only common tests should be described solely by name; describe more complex techniques in the Methods section.*
☒	☐	A description of all covariates tested
☒	☐	A description of any assumptions or corrections, such as tests of normality and adjustment for multiple comparisons
☐	☒	A full description of the statistical parameters including central tendency (e.g. means) or other basic estimates (e.g. regression coefficient) AND variation (e.g. standard deviation) or associated estimates of uncertainty (e.g. confidence intervals)
☒	☐	For null hypothesis testing, the test statistic (e.g. *F*, *t*, *r*) with confidence intervals, effect sizes, degrees of freedom and *P* value noted<br>*Give P values as exact values whenever suitable.*
☒	☐	For Bayesian analysis, information on the choice of priors and Markov chain Monte Carlo settings
☒	☐	For hierarchical and complex designs, identification of the appropriate level for tests and full reporting of outcomes
☒	☐	Estimates of effect sizes (e.g. Cohen's *d*, Pearson's *r*), indicating how they were calculated

Our web collection on statistics for biologists contains articles on many of the points above.

Software and code

Policy information about availability of computer code

Data collection	For spectroscopy measurement, FILMeasure v8 software was used for data collection.<br>For transistors and circuits characterization, Kickstart v2.4 software was used for data collection.<br>For photovoltaic performance, Enli Technology Co., Ltd. software was used for data collection.
Data analysis	For GIWAXS data, Igor v6.38B01 software was used for data analysis.<br>For AFM data, Gwyddion v2.59 software was used for data analysis.<br>Origin 2021 software was used for the analysis of other data.

For manuscripts utilizing custom algorithms or software that are central to the research but not yet described in published literature, software must be made available to editors and reviewers. We strongly encourage code deposition in a community repository (e.g. GitHub). See the Nature Portfolio guidelines for submitting code & software for further information.

Data

Policy information about availability of data

All manuscripts must include a data availability statement. This statement should provide the following information, where applicable:

- Accession codes, unique identifiers, or web links for publicly available datasets
- A description of any restrictions on data availability
- For clinical datasets or third party data, please ensure that the statement adheres to our policy

The authors declare that the main data supporting the findings of this study are available within the article and its Supplementary Information files. Source data are provided with this paper.

Research involving human participants, their data, or biological material

Policy information about studies with human participants or human data. See also policy information about sex, gender (identity/presentation), and sexual orientation and race, ethnicity and racism.

Reporting on sex and gender	This information has not been collected.
Reporting on race, ethnicity, or other socially relevant groupings	This information has not been collected.
Population characteristics	ECG, EMG and EOG samples were collected from a healthy male volunteer aged 33.
Recruitment	The first author served as the volunteer, eliminating recruitment needs, and was fully compliant with all relevant policies.
Ethics oversight	Ethics Committee of the First Affiliated Hospital of Xi'an Jiaotong University (the protocols of 2022-1203) .

Note that full information on the approval of the study protocol must also be provided in the manuscript.

Field-specific reporting

Please select the one below that is the best fit for your research. If you are not sure, read the appropriate sections before making your selection.

☒ Life sciences ☐ Behavioural & social sciences ☐ Ecological, evolutionary & environmental sciences

For a reference copy of the document with all sections, see nature.com/documents/nr-reporting-summary-flat.pdf

Life sciences study design

All studies must disclose on these points even when the disclosure is negative.

Sample size	To avoid errors caused by differences in sample information, we conducted relevant tests using the same samples for electrical physiological signal monitoring.
Data exclusions	Data were not excluded from analysis.
Replication	All organic solar cell devices and organic electrochemical transistor devices have undergone repeatability tests. For detailed information, please refer to the supporting information.
Randomization	All the organic solar cells and organic electrochemical transistor devices were not allocated into groups, thus no randomization exists. For cell culture experiment, the L-929 cells that passed quality control were analyzed equally with no sub-sampling and thus, there was no requirement for randomization. For the human research participants, the experiments were carried out by the author himself. Due to the need for privacy, randomisation was not required.
Blinding	For material, device, circuits characterizations and human research participants, the investigators were blinded to the materials, processing conditions during the data collection and analysis.

Reporting for specific materials, systems and methods

We require information from authors about some types of materials, experimental systems and methods used in many studies. Here, indicate whether each material, system or method listed is relevant to your study. If you are not sure if a list item applies to your research, read the appropriate section before selecting a response.

Materials & experimental systems

n/a	Involved in the study
x	Antibodies
	x Eukaryotic cell lines
x	Palaeontology and archaeology
x	Animals and other organisms
x	Clinical data
x	Dual use research of concern
x	Plants

Methods

n/a	Involved in the study
x	ChIP-seq
x	Flow cytometry
x	MRI-based neuroimaging

Eukaryotic cell lines

Policy information about cell lines and Sex and Gender in Research

Cell line source(s)	NCTC clone 929 (L-929) cells (rat, origin source is BeNa Culture Collection).
Authentication	Cell lines have been authenticated by vendor (BeNa Culture Collection).
Mycoplasma contamination	Cell lines were not tested for mycoplasma contamination but no indication of contamination was observed.
Commonly misidentified lines (See ICLAC register)	No commonly misidentified cell lines were used

Plants

Seed stocks	Our study does not involve any research related to plants.
Novel plant genotypes	Our study does not involve any research related to plants.
Authentication	Our study does not involve any research related to plants.
