# Supplementary material for: Light-insensitive organic solar-powered amplifiers
Source: Nat Commun. 2025 Nov 27;16:10640. doi: 10.1038/s41467-025-65640-z (PMC12660383; doi:10.1038/s41467-025-65640-z)
Supplement: Supplementary file 6 — Reporting Summary [file 41467_2025_65640_MOESM6_ESM.pdf]

nature research

Corresponding author(s): Wei Ma

Solar Cells Reporting Summary

Nature Research wishes to improve the reproducibility of the work that we publish. This form is intended for publication with all accepted papers reporting the characterization of photovoltaic devices and provides structure for consistency and transparency in reporting. Some list items might not apply to an individual manuscript, but all fields must be completed for clarity.

For further information on Nature Research policies, including our data availability policy, see Authors & Referees.

ü Experimental design

Please check: are the following details reported in the manuscript?

1. Dimensions

Item	Yes	No	Details
Area of the tested solar cells	x		The device illuminated area during testing was 0.004 cm2, which was determined by a mask. This information can be found in Method part.
Method used to determine the device area	x		The area of devices was determined by a mask.

2. Current-voltage characterization

Item	Yes	No	Details
Current density-voltage (J-V) plots in both forward and backward direction		x	The hysteresis effect of organic solar cells can be ignored, and the positive and negative scanning results are the same.
Voltage scan conditions<br>*For instance: scan direction, speed, dwell times*	x		The condition of voltage scanning is forward scanning at a speed of 0.02 V and dwell time of 1 ms.
Test environment<br>*For instance: characterization temperature, in air or in glove box*	x		The devices were characterized at room temperature in a glove box.
Protocol for preconditioning of the device before its characterization		x	No preconditioning protocol.
Stability of the J-V characteristic<br>*Verified with time evolution of the maximum power point or with the photocurrent at maximum power point; see ref. 7 for details.*	x		We tested the light and bending stabilities in our lab.

3. Hysteresis or any other unusual behaviour

Item	Yes	No	Details
Description of the unusual behaviour observed during the characterization		x	No hysteresis or other unusual behaviour was observed during the characterization of the solar cells. In general, organic solar cells do not have hysteresis problems.
Related experimental data		x	No hysteresis or other unusual behaviour was observed during the characterization of the solar cells.

4. Efficiency

Item	Yes	No	Details
External quantum efficiency (EQE) or incident photons to current efficiency (IPCE)	x		This information can be found in manuscript .
A comparison between the integrated response under the standard reference spectrum and the response measure under the simulator	x		This information can be found in OSCs fabrication and characterization section.
For tandem solar cells, the bias illumination and bias voltage used for each subcell		x	No tandem solar cell was reported in this manuscript.

5. Calibration

Item	Yes	No	Details
Light source and reference cell or sensor used for the characterization	x		Enli Technology AAA solar simulator (SS-FS) and standard Si (SRC-2020) reference cell were used during solar cells testing (Fabrication and measurement of OSCs devices)
Confirmation that the reference cell was calibrated and certified	x		This information can be found in OSCs fabrication and characterization section.

Calculation of spectral mismatch between the reference cell and the devices under test

- [ ] Yes
- [x] No

No spectral mismatch calculation was performed in our lab.

6. Mask/aperture

Size of the mask/aperture used during testing

- [x] Yes
- [ ] No

This information can be found in OSCs fabrication and characterization section.

Variation of the measured short-circuit current density with the mask/aperture area

- [ ] Yes
- [x] No

We measured all devices with the same mask.

7. Performance certification

Identity of the independent certification laboratory that confirmed the photovoltaic performance

- [ ] Yes
- [x] No

Explain why this information is not reported/not relevant.

A copy of any certificate(s)
Provide in Supplementary Information

- [ ] Yes
- [x] No

Explain why this information is not reported/not relevant.

8. Statistics

Number of solar cells tested

- [x] Yes
- [ ] No

12 devices. This information can be found in manuscript and Supplementary Information.

Statistical analysis of the device performance

- [x] Yes
- [ ] No

The average PCE were calculated from 12 devices for each OSC. This information can be found in manuscript and Supplementary Information.

9. Long-term stability analysis

Type of analysis, bias conditions and environmental conditions
For instance: illumination type, temperature, atmosphere humidity, encapsulation method, preconditioning temperature

- [x] Yes
- [ ] No

This information can be found in OSCs fabrication and characterization section in Supplementary Information.
